# Bayesian adaptive decision-theoretic designs for multi-arm multi-stage clinical trials

**DOI:** 10.1177/0962280220973697

**Published:** 2020-11-26

**Authors:** Andrea Bassi, Johannes Berkhof, Daphne de Jong, Peter M van de Ven

**Affiliations:** 1 Department of Epidemiology and Data Science, Amsterdam UMC, Vrije Universiteit Amsterdam, Amsterdam, The Netherlands; 2 Department of Pathology, Amsterdam UMC, Vrije Universiteit Amsterdam, Amsterdam, The Netherlands

**Keywords:** Adaptive design, Bayesian, clinical trials, decision theory, multi-arm multi-stage trials

## Abstract

Multi-arm multi-stage clinical trials in which more than two drugs are simultaneously investigated provide gains over separate single- or two-arm trials. In this paper we propose a generic Bayesian adaptive decision-theoretic design for multi-arm multi-stage clinical trials with *K* (K≥2) arms. The basic idea is that after each stage a decision about continuation of the trial and accrual of patients for an additional stage is made on the basis of the expected reduction in loss. For this purpose, we define a loss function that incorporates the patient accrual costs as well as costs associated with an incorrect decision at the end of the trial. An attractive feature of our loss function is that its estimation is computationally undemanding, also when *K *>* *2. We evaluate the frequentist operating characteristics for settings with a binary outcome and multiple experimental arms. We consider both the situation with and without a control arm. In a simulation study, we show that our design increases the probability of making a correct decision at the end of the trial as compared to nonadaptive designs and adaptive two-stage designs.

## 1. Introduction

Modern medicine has seen a rapid increase in the number of drugs on the market. The efficacy of a drug is traditionally evaluated in single-arm or two-arm trials. Trials with more than two arms are increasingly demanded and are particularly suited when multiple, competing drugs are being developed or combinations of drugs are being tested.^1,2^ Also, in the current COVID-19 pandemic there is an urgent need to investigate multiple treatments simultaneously. The RECOVERY trial, for example, compares efficacy of four candidate treatments for COVID-19 to a common control arm receiving usual care.^3^ Trials with more than two arms typically require fewer overall resources than multiple two-arm trials and facilitate a direct comparison of drugs.^4–7^ In this paper, we study trials in which one or a few drugs are selected from a set of candidate drugs. The selected drugs may enter the next trial phase or may be proposed for approval. During the trial, it is desirable to select or deselect drugs as soon as possible. Timely decision making is facilitated by incorporating interim evaluations.^8–10^ We will refer to trials in which patients are randomized over multiple arms with multiple interim evaluations as multi-arm multi-stage (MAMS) trials. MAMS typically allow early termination of ineffective arms and early identification of effective arms.

Frequentist MAMS trials are characterized by repeated statistical tests of futility and efficacy null hypotheses. The critical boundaries are set such that the familywise error rate over the whole trial is controlled at a predefined nominal level.^2,8,9,11–13^ Experimental arms are compared to the control arm and each experimental arm may be declared effective or futile at each interim evaluation. Bayesian MAMS use a predefined stopping rule based on the posterior distribution of a function of the treatment efficacies.^14^ Bayesian designs often involve response-adaptive randomization where randomization probabilities change throughout the trial based on newly collected treatment outcomes.^14,15^ A response-adaptive design will lead to a higher expected number of patients allocated to the best arm during the trial in comparison to trials with an equal randomization scheme and may therefore be attractive for ethical reasons. However, this may come at a price of lower statistical power for showing differences in efficacy between the arms.^16^

Stopping rules in Bayesian MAMS trials may be based on direct, simple functions of the treatment efficacies, but more formal decision-theoretic approaches also exist.^17–27^ Decision-theoretic approaches quantify the value of all possible trial outcomes by means of a loss or utility function. The objective of the trial is to minimize the expected loss of the trial or to maximize the expected utility. A clever choice of the loss function may yield Bayesian trials with lower expected trial sizes than classical frequentist trials at the same nominal error rate.^17^ Most Bayesian decision-theoretic designs have been proposed for trials with two arms. If the trial has a predefined maximum study size, then at each stage the optimal interim decision needs to be assessed by a computationally expensive dynamic programming approach.^17–19,21–23,25,27^ Several strategies have been proposed in the literature to limit the computational burden. Cheng and Shen^20^ did not fix the maximum trial size before the start of the trial and propose a one-step backward induction algorithm. They showed that their study design will always result in a finite trial size. Jiang et al.^26^ proposed a constrained backward induction algorithm on a reduced lower-dimensional state space to approximate the optimal stop or continue decision at each stage. Both Cheng and Shen^20^ and Jiang et al.^26^ studied two-arm trials only. Orawo and Christen^24^ extended to the general situation of trials with *K* (K≥2) arms and aimed to select the best arm. They proposed a stopping rule for the *K*-arm trial based on optimal stopping rules in single-arm trials. After each new observation, the trial is either stopped and the best arm is selected or an additional patient is added to the arm that showed the best performance so far.

Willan and Kowgier^23^ and Chen and Willan^25^ considered multi-stage adaptive designs from a value of information perspective.^28^ They did not fix the maximum total sample size, but fixed the maximum number of stages. They considered the expected net gain of the trial, which is defined as the difference between the expected value of sample information and the total costs of the trial. At each stage, they determine an optimal sample size for the remainder of the trial and the fraction of the sample size that needs to be recruited in the next stage through maximization of the expected net gain. Their method is computationally demanding and has only been developed for trials of two stages.

The one-step backward induction algorithm of Cheng and Shen^20^ also has a link to value of information considerations. After each interim evaluation, Cheng and Shen compared the cost of continuing the trial for one more stage with the expected reduction in loss. If the extra costs exceed the expected loss reduction, the trial is stopped and the arm with the lowest expected loss is selected. Stated differently, if the expected marginal utility (or loss reduction) of continuing the trial does not exceed the marginal cost of continuing the trial, the trial is stopped. The approach is generic in the sense that any loss function can be defined and a different loss function can be defined for the experimental and control arm.

In the present study, we generalize the framework introduced by Cheng and Shen to the setting with *K* (K≥2) arms. Since more than two arms are allowed, a trialist may decide to stop ineffective, futile arm(s) and continue the trial with only a subset of study arms. In our generalization, we also consider such trial-modifying decisions that do not apply to the setting with only two arms. The main objective of the trial is to find the best arm among *K* experimental arms or, in settings with a control arm, identify all experimental arms that outperform the control arm. A key evaluation measure in our study design is the probability of making a correct final decision. The decision to stop or continue the trial is directly based on the expected increase in this probability when continuing for an additional stage. The repeated interim evaluations can be seen as a series of expected net gain assessments. The trial continues as long as the expected value of sample information provided by a next stage of patients exceeds the costs of their accrual. An attractive feature of our method is that the estimation of the expected loss reduction is computationally undemanding, also in settings with *K *>* *2 arms.

This paper is organized as follows. In the following section, we describe the Bayesian decision-theoretic framework and provide examples of loss functions. Next, we describe two simulation studies in which we evaluate the operating characteristics of our approach and make a comparison with nonadaptive single-stage and adaptive two-stage methods. In the simulations, we consider multi-arm trials with and without a control group. The results of the simulations are discussed in a later section. Finally, we show an illustration of a possible application of the methods in a future trial and we conclude with a discussion in the last section.

## 2. Methods

### 2.1. Notation

We consider the general setting of a MAMS trial with *K* (K≥2) arms where at the end of each stage ineffective arms may be dropped from the trial. An arm that is in the trial is called an active arm and a dropped arm is called an inactive arm. A predetermined number of *n*_1_ patients are recruited to the first stage of the trial followed by *n* patients in each of the subsequent stages. Allowing *n*_1_ to be larger than *n* prevents early termination based on a limited number of observed outcomes. The patients that are recruited in a single stage are randomly assigned to the active arms in equal numbers. We assume patients’ treatment outcomes *y* to be independent and denote the likelihood of the treatment outcome of a patient in arm *k* by $f\left(y|\theta_{k}\right)$, where *θ_k_* is a scalar. The parameters *θ_k_* (k=1,…,K) are assumed to have independent prior distributions $p(\theta_{k})$. The distribution of the treatment outcomes may depend on other unknown parameters, but we will suppress reference to these parameters for ease of notation. We let the random variable *Y_ik_* denote the vector of treatment outcomes for the patients that in stage *i* are assigned to arm *k*. The accumulated data up to stage *s* is denoted by $Y_{s}=\{Y_{ik}|1\leq i\leq s,1\leq k\leq K\}$.

### 2.2. Loss function and decision rules

Suppose we have a study design where all arms are active until a final decision is made. The predefined set of final decisions is denoted by *D*. The loss of each final decision depends on the unknown parameter vector $\theta =\left(\theta_{1},\ldots ,\theta_{K}\right)$. By L(θ,Q,d) we denote the loss associated with decision d∈D where *Q* denotes the loss associated with an incorrect decision at the end of the trial. In line with Pratt et al.^29^ and Cheng and Shen,^20^ we define the expected total loss in case of terminating the trial after stage *s* as

$$L_{\text{stop}}\left(Y_{s}\right)=C_{s}+{\text{min}}_{d\in D}\;\text{E}\left[L\left(\theta ,Q,d\right)|Y_{s}\right],$$

where *C_s_* are the total costs of running the first *s* stages of the trial. The expected total loss in case of continuing the trial for an additional stage is

$$L_{\text{cont}}\left(Y_{s}\right)=C_{s+1}+\text{E}\left[{\text{min}}_{d\in D}\;\text{E}\left[L\left(\theta ,Q,d\right)|Y_{s+1}\right]|Y_{s}\right],$$

where the outside expectation is taken with respect to the posterior predictive distribution of $Y_{\left(s+1\right)1},\ldots ,Y_{\left(s+1\right)k}$ given $Y_{s}$ and taking into account the prespecified sample size *n* for stage *s *+* *1. The decision to continue is made by comparing the expected total loss in case of stopping with the expected total loss in case of continuing the trial for one more stage. In case $L_{\text{stop}}\leq L_{\text{cont}}$, the trial is stopped and the final decision $d^{*}$ is made, where

$$d^{*}={\text{argmin}}_{d\in D}\text{E}\left[L\left(\theta ,Q,d\right)|Y_{s}\right]$$

If $L_{\text{stop}}>L_{\text{cont}}$, then the additional *n* patients will be accrued and a new decision regarding continuation will be made at the end of stage *s *+* *1.

We now consider the possibility of early dropping of ineffective arms and, hence, in case of continuation an additional decision needs to be made on how to continue the trial. To describe a design with early dropping, we introduce $M_{s+1}$ as the set of different options for stage *s *+* *1 of the trial. Then the expected total loss in case of continuation is

(1) $$L_{\text{cont}}\left(Y_{s}\right)=C_{s+1}+\min_{m\in M_{s+1}}\text{E}\left[{\text{min}}_{d\in D}\;\text{E}\left[L\left(\theta ,Q,d\right)|Y_{s+1}^{m}\right]|Y_{s}\right]$$

where $\text{E}\left[L\left(\theta ,Q,d\right)|Y_{s+1}^{m}\right]$ is the expected loss incurred by the final decision conditional on data $Y_{s+1}^{m}$ accumulated up to stage *s *+* *1, where the superscript refers to the choice for $m\in M_{s+1}$. The outside expectation in (1) is again taken with respect to the posterior predictive distribution of $Y_{\left(s+1\right)1}^{m},\;\ldots ,\;Y_{\left(s+1\right)k}^{m}$ given $Y_{s}$ taking sample size *n* and option $m\in M_{s+1}$ into account. The loss function L(θ,Q,d) and set of final decisions *D* are not affected by decisions in $M_{s+1}$ and remain unchanged throughout the trial.

### 2.3. Examples

*Select the best arm from K experimental arms.* We define the set of final decisions as $D=\{d_{1},d_{2},\ldots ,d_{K}\}$, where *d_k_* refers to the decision to select arm *k*. If we assume that higher values of *θ_k_* indicate higher efficacy and lower loss, then a possible loss function is

(2) $$L\left(\theta ,Q,d_{k}\right)=\{\begin{array}{l} 0\text{if }\theta_{k}=\max_{i:1\leq i\leq K}\theta_{i};\\ Q\text{otherwise} \end{array}$$

in which case the expected loss $\text{E}\left[L\left(\theta ,d_{k}\right)|Y_{s}\right]$ is equal to the posterior probability that arm *k* is not the best performing arm multiplied by the loss *Q* for an incorrect decision.

If early dropping of ineffective arms is allowed in a setting with *K *>* *2, options for continuing the trial also need to be defined. One may, for instance, adopt a strategy where at each interim analysis at most one arm is dropped from the trial. In case of *K *=* *3 arms this is done by defining $M_{2}=\{m_{123},m_{12},m_{13},m_{23}\}$, where *m*_123_ denotes the decision to retain all arms in the next stage of the trial and *m*_12_, *m*_13_ and *m*_23_ denote the decisions to drop arm 3, 2, and 1, respectively. As long as *m*_123_ is selected in subsequent stages, all arms are retained in the trial and $M_{s+1}:=M_{s}$.

*Select the unique best arm from K experimental arms in the presence of an equivalence margin*. If one wants to avoid that a best arm needs to be selected in case multiple arms show similar performance, then an equivalence margin can be incorporated in the loss function. We assume that one wants to select the best arm only if it outperforms the other arms by a prespecified margin δ>0. If no such arm exists then none of the arms should be selected. We extend the set of final decisions from the previous example to $D=\{d_{\emptyset },d_{1},d_{2},\ldots ,d_{K}\}$, where $d_{\emptyset }$ refers to the decision that there exists no unique best arm. The loss function L(θ,Q,d) is

$$\{\begin{array}{l} 0\text{if }d=d_{k}\text{ and }\theta_{k}-\theta_{j}>\delta \text{ for all }j\in \{1,\ldots ,K\}\setminus \{k\};\\ 0\text{if }d=d_{\emptyset }\text{ and }\max_{i:1\leq i\leq K}\theta_{i}-\theta_{j}\leq \delta \text{ for some }j\in \{1,\ldots ,K\}\setminus \{{\text{argmax}}_{i}\;\theta_{i}\};\\ Q\text{otherwise} \end{array}$$

*Select the T best arms from K experimental arms.* Especially if *K* is large, the goal may be to select a predefined number of *T* (*T *<* K*) most promising experimental arms that warrant further investigation. If, for instance, one aims to select the two best arms, then the final decisions are $D=\{d_{jk}|1\leq j<k\leq K\}$, where *d_jk_* refers to the decision of selecting arm *j* and *k*. A possible loss function is

$$L\left(\theta ,Q,d_{jk}\right)=\{\begin{array}{l} 0\text{if }\left(\theta_{j}+\theta_{k}\right)=\max_{1\leq p<q\leq K}\left(\theta_{p}+\theta_{q}\right);\\ Q\text{otherwise} \end{array}$$

*Compare K – 1 experimental arms to a control arm.* We assume that *θ*_1_ refers to the parameter of interest in the control arm. In the presence of a control arm and multiple experimental arms the aim typically is to identify all the experimental arms that outperform the control arm. If, for instance, *K *=* *3, then we define $D=\{d_{1},d_{2},d_{3},d_{23}\}$, where *d*_1_ refers to the decision to declare none of the experimental arms superior to control. Decisions *d*_2_ and *d*_3_ correspond to declaring only experimental arms 2 and 3 superior to the control arm, respectively. Decision *d*_23_ refers to declaring both experimental arms superior to the control arm. We denote by *δ* the prespecified superiority margin for the difference between experimental treatments and control. If we set the expected loss proportional to the posterior probability of an incorrect decision, then the loss function is

(3) $$L\left(\theta ,Q,d\right)=\{\begin{array}{l} 0\text{if }d=d_{1},\theta_{2}\leq \theta_{1}+\delta \text{ and }\theta_{3}\leq \theta_{1}+\delta ;\\ 0\text{if }d=d_{2},\theta_{2}>\theta_{1}+\delta \text{ and }\theta_{3}\leq \theta_{1}+\delta ;\\ 0\text{if }d=d_{3},\theta_{2}\leq \theta_{1}+\delta \text{ and }\theta_{3}>\theta_{1}+\delta ;\\ 0\text{if }d=d_{23},\theta_{2}>\theta_{1}+\delta \text{ and }\theta_{3}>\theta_{1}+\delta ;\\ Q\text{otherwise} \end{array}$$

### 3. Simulation studies

We evaluate the frequentist operating characteristics of the Bayesian decision-theoretic designs in trials with multiple arms and a binary outcome. We consider the setting without and with a control arm. In simulation I, we consider selection of the best experimental arm from *K* (*K *=* *3, 4, 5) experimental arms using loss function (2). In simulation II, we compare two experimental arms to a common control arm using loss function (3). In both simulation studies, we let each *Y_ik_* (i=1,2,…,s and k=1,2,…,K) contain independent observations from a Bernoulli distribution with success probability *θ_k_*. We assume independent priors $p(\theta_{k})\propto U\left(0,1\right)$. We assume $C_{s}=C_{1}+(s-1)C$, which corresponds to equal costs for all stages with a possible exception for the initial stage. We set the simulation size at 5,000 trials per setting. Further details on the design of the simulation studies follow below. R functions for evaluating the frequentist operating characteristics are provided in a github repository (see Appendix 1).

#### Simulation I: Three to five experimental arms

In simulation I, we compare three Bayesian decision-theoretic designs B1, B2, and B3. In all three designs, the final decision is based on minimization of the posterior expected loss. Design B1 is a MAMS trial with adaptive stopping and the possibility of early dropping of ineffective arms from the trial. After each stage, it is decided whether the trial continues with the same arms, a single arm is dropped or the trial is stopped. The trial is stopped after stage *s* if

(4) $${\text{min}}_{d\in D}\;\text{E}\left[L\left(\theta ,Q,d\right)|Y_{s}\right]-$$

$$\underset{{\;}_{m\in M_{s+1}}}{\min}\text{E}\left[{\text{min}}_{d\in D}\;\text{E}\left[L\left(\theta ,Q,d\right)|Y_{s+1}^{m}\right]|Y_{s}\right]\leq C$$

where $M_{s+1}$ denotes the set of available options for stage *s *+* *1 of the trial. Design B2 is a MAMS trial with adaptive stopping in which all *K* arms are retained until the end of the trial. This trial is stopped after stage *s* if

(5) $${\text{min}}_{d\in D}\;\text{E}\left[L\left(\theta ,Q,d\right)|Y_{s}\right]-\text{E}\left[{\text{min}}_{d\in D}\;\text{E}\left[L\left(\theta ,Q,d\right)|Y_{s+1}\right]|Y_{s}\right]\leq C$$

Design B3 is a single-stage, nonadaptive trial (with fixed trial size) in which patients are allocated to the *K* arms in equal numbers.

This simulation study consists of three substudies, which we denote by I.1–3. In simulation I.1, we compare B1 and B2 for settings with *K *=* *3 to 5 arms and report the proportion of correct decisions and average trial sizes. We set the sizes for the initial batch and subsequent batches at 4 *K*, i.e. $n_{1}=n=12$ for *K *=* *3, $n_{1}=n=16$ for *K *=* *4 and $n_{1}=n=20$ for *K *=* *5. Low values were chosen for *n*_1_ and *n* to facilitate timely stopping of the trials in case large differences exist between response rates. For *K *=* *3, we set $\theta =(\theta_{1},\theta_{2},\theta_{3})$ equal to (0.2, 0.6, 0.7), (0.2, 0.7, 0.8), (0.2, 0.8, 0.9), (0.5, 0.5, 0.6), (0.5, 0.5, 0.7), (0.5, 0.5, 0.8), (0.5, 0.6, 0.7), (0.5, 0.7, 0.8), and (0.5, 0.8, 0.9). We extend *K *=* *3 to *K *=* *4 and *K *=* *5 by setting *θ* equal to $\left(\theta_{1},\theta_{2},\theta_{3},\theta_{1}\right)$ and $\left(\theta_{1},\theta_{2},\theta_{3},\theta_{1},\theta_{2}\right)$. If we divide both the left- and right-hand side of (4) and (5) by *Q*, it follows for loss functions (2) and (3) that the trials of design B1 and B2 are stopped as soon as the expected increase in the posterior probability of making a correct decision drops below *C*/*Q*. In simulation I.1, we set *C*/*Q* equal to 1/500, 1/1000, 1/2500, 1/5000 and 1/10,000.

In simulation I.2, we compare B1 and B2 to nonadaptive B3 in terms of the proportion of correct decisions after equalizing the average sample size of the three designs. More specifically, we start by setting *C*/*Q* for design B1 equal to 1/2500 and run simulations under B1. Then, for design B2 we determine separately for each setting of *θ* the value of *C*/*Q* for which the average trial size is equal to that observed under design B1. Similarly, for design B3, we use the trial sizes obtained under design B1. We confine ourselves to *K *=* *3 and set batch size and $\theta =(\theta_{1},\theta_{2},\theta_{3})$ equal to those in simulation I.1. We repeat the simulation comparing the average trial sizes of designs B2 and B3 with those of B1 after equalizing the proportion of correct decisions.

Finally, in simulation I.3, we study the frequentist operating characteristics of the relative costs *C*/*Q*. We set *C*/*Q* equal to 1/100,1/250,1/1000, and 1/2500 and simulate under design B2. For each of the simulated trials, we simulate one extra stage and calculate the increase in the proportion of trials with a correct decision after one more stage. We only consider *K *=* *3 and set batch sizes and $\theta =(\theta_{1},\theta_{2},\theta_{3})$ equal to those in simulation I.1.

#### Simulation II: Two experimental arms and a control arm

In simulation II, we compare design B2 to three frequentist designs F1, F2, and F3. Design F1 is a single-stage, nonadaptive trial with fixed trial size and equal allocation of patients to arms. The final decision under F1 is based on the outcome of Dunnett’s multiple comparison hypothesis testing procedure for comparing multiple experimental arms to a control while controlling the familywise type I error rate. Design F2 and F3 are adaptive two-stage designs using the closed testing procedure of Urach and Posch^9^ with equal allocation of patients to the active arms in both stages. Both F2 and F3 include an interim analysis after half of the maximum number of patients have been enrolled. We use arm-specific stopping rules where in the interim analysis each individual experimental arm can be declared futile or superior to the control after which accrual is stopped for that arm. Both designs F2 and F3 declare an arm futile when the interim Z test statistic is negative. For concluding superiority, design F2 uses O’Brien Fleming-type boundaries whereas F3 uses Pocock-type boundaries. We set the one-sided type I error rate at 5%. We assume that arm 1 is the control arm and that arms 2 and 3 are experimental arms. We set *θ* equal to: (0.5, 0.5, 0.7), (0.5, 0.7, 0.7), (0.5, 0.5, 0.8), (0.5, 0.7, 0.8), and (0.5, 0.8, 0.8). We set margin *δ* in loss function (3) equal to 0.15. For this margin, the decision that minimizes loss (3) is equal to *d*_3_ for θ=(0.5,0.5,0.7) or (0.5,0.5,0.8), and equal to *d*_23_ for θ=(0.5,0.7,0.7),(0.5,0.7,0.8), or (0.5,0.8,0.8). We set the initial and subsequent batch sizes under B2 to $n_{1}=24$ and *n *=* *12, respectively. The size of 24 for the initial stage was selected because it was the smallest multiple of 12 for which the familywise type I error rate could be controlled at the target level. We control the familywise type I error under B2 by tuning of *C*/*Q*. This is done by selecting the value of *C*/*Q* for which the control arm is selected in 95% of the trials simulated under B2 when θ=(0.5,0.5,0.5). This provides a conservative testing procedure as the posterior variance is largest when the success probabilities are equal to 0.5. For each $\theta =\left(\theta_{1},\theta_{2},\theta_{3}\right)$, we first simulate under B2 and subsequently set trial sizes for F1 equal to the trial sizes observed under B2. We then simulate trials under F2 and F3 and increase the maximum trial size until the average trial size equals that observed under B2. We compare the four designs with respect to the proportion of trials with a correct decision. We repeat the simulation comparing the average trial sizes of designs F1, F2, and F3 with those of B2 after equalizing the proportion of correct decisions under the hypothesized response rates $\theta =\left(\theta_{1},\theta_{2},\theta_{3}\right)$. More specifically, we compare the four designs with respect to their average sample size when the true response rates equal the hypothesized response rates $\theta =\left(\theta_{1},\theta_{2},\theta_{3}\right)$ and when the true response rates equal the null response rates θ=(0.5,0.5,0.5).

### 4. Simulation results

**Figure 1.**
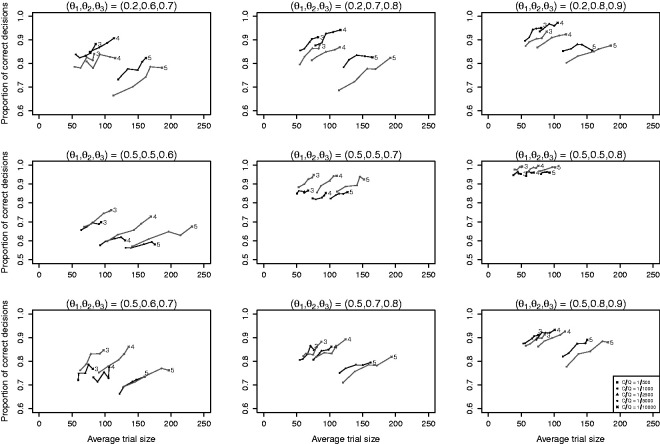
Results of simulation I.1 with *K *=* *3, 4, 5 experimental arms. Line segments connect results for the same design and number of arms for different relative costs *C*/*Q*. Black lines are used for design B1 (Bayesian MAMS with dropping) and grey lines for design B2 (Bayesian MAMS without dropping). The numbers at the end of the lines denote the number of arms *K*.

Results of simulation I.1 are presented in Figure 1 where each panel corresponds with a setting of *θ* and lines connect results for the same design under different relative costs *C*/*Q*. The proportion of correct decisions is at least 0.70 for most settings, except for the left panel in the middle row. Average trial sizes do not exceed 250 and are below 150 for the majority of settings. In the top row panels where the success probabilities differ at least 0.4 between the worst and other arms, average trial sizes are similar under B1 and B2, but proportions of correct decisions are higher under B1. An explanation for this is that design B1 adapts during the trial by allocating more patients to the best performing arms, thereby providing more information for identification of the best performing arm. In the middle row panels where all arms have the same success probability except for the best performing arm, we see that average trial sizes decrease and proportions of correct decisions increase when the success probability of the best performing arm increases. If the best arm is clearly superior to the other arms (middle row, right panel), then the average trial size remains below 100 for most of the settings. The proportion of correct decisions is slightly higher for B2 than for B1 at similar average trial sizes indicating that early dropping may negatively influence the performance of the trial when there is no clearly inferior treatment arm. In the bottom row panels where differences in success probability between the worst and other arms are smaller than in the upper row panels, we see that B1 and B2 perform similarly in terms of both average trial size and proportion of correct decisions.

When comparing the three rows, we see that the average trial size under both B1 and B2 depends strongly on the difference between the best and second-best arm (middle row), but only weakly on the difference between the worst and other arms (top versus bottom row). We also see that the effect of adding an extra arm on the average trial size depends on the success probability of the arm that is added. When the success probabilities of the added arms are equal to that of the worst arm (middle row), the average trial size increases approximately linearly with the number of arms. However, increases in average trials size were found to be larger when the success probability of the added arm was closer to that of the best arm as this made it more difficult to select the best arm (top and bottom row).

**Figure 2.**
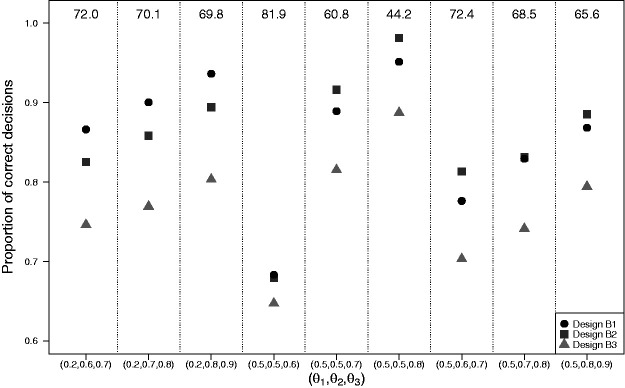
Results of simulation I.2 with *K *=* *3 experimental arms with the average trial size equalized across designs. Matching of the designs in terms of the average trial size was done separately for each setting of $\theta =\left(\theta_{1},\theta_{2},\theta_{3}\right)$. Average trial sizes are given in the upper region. Design B1: Bayesian decision-theoretic MAMS trial with early dropping and adaptive stopping; Design B2: Bayesian decision-theoretic MAMS trial without early dropping but with adaptive stopping; Design B3: Single-stage trial with fixed predefined trial size.

Figure 2 shows the results of simulation I.2 with the average trial size equalized across designs. Designs with adaptive stopping (designs B1 and B2) outperform the single-stage trial design B3 in terms of the proportion of correct decisions by 5% to 15%. The added value of early dropping depends on *θ* in accordance with what we observed in simulation I.1. Table 1 shows average trial sizes after equalizing the proportion of correct decisions. We see that average trial sizes of B2 are 26% to 33% higher than under B1 when $\theta_{1}=0.2$. Trial sizes of B3 are increased by 117% to 157% as compared to B1. For the scenarios with $\theta_{1}=0.5$, average trial sizes of B2 are reduced by 2% to 27% when compared to B1. Trial sizes of B3 are increased by 16% to 58% as compared to B1.

**Table 1.** Results of simulation I.2 with *K *=* *3 experimental arms with the proportion of correct decisions equalized across designs. Matching of the designs in terms of the proportion of correct decisions was done separately for each setting of $\theta =\left(\theta_{1},\theta_{2},\theta_{3}\right)$. Design B1: Bayesian decision-theoretic MAMS trial with early dropping and adaptive stopping; Design B2: Bayesian decision-theoretic MAMS trial without early dropping but with adaptive stopping; Design B3: Single-stage trial with fixed predefined trial size.

Response rate vector	Proportion of correct decisions	Average trial size		
$\left(\theta_{1},\theta_{2},\theta_{3}\right)$		B1	B2	B3
(0.2,0.6,0.7)	0.87	72.0	90.6	156
(0.2,0.7,0.8)	0.90	70.1	92.9	180
(0.2,0.8,0.9)	0.94	69.8	88.2	162
(0.5,0.5,0.6)	0.68	81.9	72.3	96
(0.5,0.5,0.7)	0.89	60.8	53.2	84
(0.5,0.5,0.8)	0.95	44.2	32.3	54
(0.5,0.6,0.7)	0.78	72.4	61.4	84
(0.5,0.7,0.8)	0.83	68.5	67.7	108
(0.5,0.8,0.9)	0.87	65.6	57.5	99

**Figure 3.**
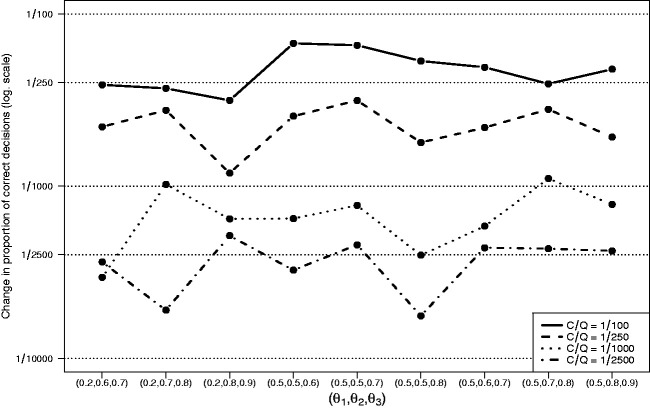
Results of simulation I.3 with *K *=* *3 experimental arms. The change in proportion of trials with a correct decision when trials continue for a single additional stage after a decision to stop has been taken.

Figure 3 shows the results of simulation I.3. For all settings *θ*, the average change in the proportion of trials with a correct decision is slightly lower or approximately equal to the relative costs *C*/*Q*. Therefore, stopping based on (5) supports the frequentist interpretation of *C*/*Q* as a threshold for the average increase in the proportion of trials with a correct decision.

**Figure 4.**
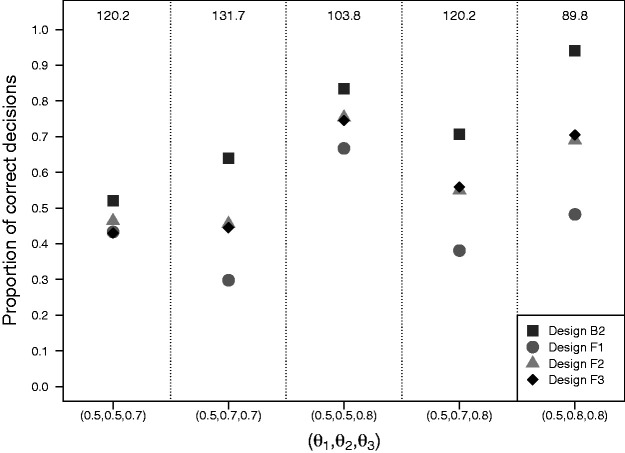
Results of simulation II with two experimental arms and a control arm with the average trial size equalized across designs. Matching of the designs in terms of the average trial size was done separately for each setting of $\theta =\left(\theta_{1},\theta_{2},\theta_{3}\right)$. Average trial sizes are given in the upper region. Design B2: Bayesian decision-theoretic MAMS with adaptive stopping; Design F1: Single-stage trial with fixed predefined trial size using Dunnett’s test; Design F2 and F3: Two-stage trials with fixed predefined maximum trial size using Urach and Posch method with O’Brien Fleming and Pocock-type boundaries, respectively.

Figure 4 presents the results of simulation II with the average trial size equalized for the four designs. Design B2 performs best and design F1 (Dunnett’s frequentist procedure) performs worst under all scenarios. Designs F2 and F3 (both Urach and Posch’s frequentist procedure) generally perform better than F1, but worse than B2. The difference in performance between design B2 and designs F1, F2 and F3 depends on *θ* and is more pronounced when both experimental arms are superior to the control arm. Under those scenarios, the difference in the proportion of correct decisions can reach 50% for designs B2 and F1 and 25% for design B2 and designs F2 and F3. Under design B2, the proportion of correct decisions regarding superiority of experimental arm *j* (*j *=* *2, 3) depends on the difference between *θ_j_* and the threshold $\theta_{1}+\delta$ for superiority. Under design F1, the proportion of correct decisions depends on the type I and type II error probabilities of the individual hypotheses for comparison of each experimental arm to control. The lower proportion of correct decisions observed for design F1 in settings where both experimental arms are superior to control are most likely the result of the type II error probabilities for each of the individual hypotheses being much higher than the type I error probabilities, so that it is more likely to make a correct decision under $\theta_{1}=\theta_{2}<\theta_{3}$ than under $\theta_{1}<\theta_{2}=\theta_{3}$. Designs F2 and F3 clearly outperform design F1 when both experimental arms are superior. Table 2 shows average trial sizes after equalizing the proportion of correct decisions. When the true response rates equal the hypothesized response rates, we find trial sizes under F1 to be increased by 22% to 120% when compared to B2. Under F2 and F3 average trial sizes are increased by 14% to 70% and 22% to 45% when compared to B2. Also when new trials are simulated under the null response rate vector θ=(0.5,0.5,0.5) instead of the hypothesized response rate vector $\theta =\left(\theta_{1},\theta_{2},\theta_{3}\right)$, we find average trial sizes under all three frequentist designs F1, F2, and F3 to be higher than under B2.

**Table 2.** Results of simulation II with two experimental arms and a control arm with the proportion of correct decisions equalized across designs. Matching of the designs in terms of the proportion of correct decisions was done separately for each hypothesized response rate vector. Average trial sizes were determined when simulating new trial data under the hypothesized response rate vector $\theta =\left(\theta_{1},\theta_{2},\theta_{3}\right)$ and the null response rate vector θ=(0.5,0.5,0.5). Design B2: Bayesian decision-theoretic MAMS with adaptive stopping; Design F1: Single-stage trial with fixed predefined trial size using Dunnett’s test; Design F2 and F3: Two-stage trials with fixed predefined maximum trial size using Urach and Posch method with O’Brien Fleming and Pocock-type boundaries, respectively.

Hypothesized response rate vector	True response rate vector	Proportion of correct decisions	Average trial size			
			B2	F1	F2	F3
(0.5,0.5,0.7)	(0.5,0.5,0.7)	0.52	120.2	147	137.5	150.9
	(0.5,0.5,0.5)	0.95	84.4	147	120.9	139.4
(0.5,0.7,0.7)	(0.5, 0.7, 0.7)	0.63	131.7	249	192.5	190.7
	(0.5,0.5,0.5)	0.95	84.4	249	158.9	180.6
(0.5,0.5,0.8)	(0.5,0.5,0.8)	0.83	103.8	129	127.7	126.4
	(0.5,0.5,0.5)	0.95	84.4	129	111.9	124.7
(0.5,0.7,0.8)	(0.5,0.7,0.8)	0.71	120.2	219	159.4	148.9
	(0.5,0.5,0.5)	0.95	84.4	219	134.6	153.5
(0.5,0.8,0.8)	(0.5,0.8,0.8)	0.94	89.8	198	152.7	129.6
	(0.5,0.5,0.5)	0.95	84.4	198	135.2	153.3

### 5. Practical example

Physical exercise programs have been shown to be effective in improving quality of life and physical functioning in patients with cancer.^30^ Recently, it has been suggested that physical exercise programs during chemotherapy may also improve response to chemotherapy.^31^ We designed a Bayesian decision-theoretic MAMS trial to compare two different physical exercise programs (resistance and aerobic exercise) to usual care in breast cancer patients receiving neoadjuvant chemotherapy. The primary outcome was tumor response defined as absence of invasive or noninvasive residual tumor after chemotherapy. The response rate under usual care was known to be around 0.20. A new exercise program for future patients and usual care were regarded equipoised when the exercise program improved tumor response rate by 0.10. An improvement of at least 0.15 was expected and was considered clinically relevant. In order to avoid exposing too many patients to ineffective exercise programs during the already burdensome chemotherapy, it was considered important that experimental arms could be dropped from the trial early if proven futile. A maximum number of 400 patients could be accrued to the trial.

We used loss function (3) with margin *δ* set at 0.10. Although the trial would be fully analyzed using Bayesian methods, adequate frequentist properties were desired. We defined the null scenario as θ=(0.20,0.20,0.20) and the alternative scenario as θ=(0.20,0.20,0.35). It was desired that in 95% of trials under the null scenario neither of the experimental arms was declared superior to control, corresponding to a frequentist one-sided familywise type I error rate of 5%. Additionally, under the alternative scenario, 80% of trials should declare arm 3 superior to the control (either alone or in combination with arm 2), corresponding to a type II error rate of 20%. It was decided to set the batch size *n* at 36. The relative loss *C*/*Q* and the size *n*_1_ of the initial stage were considered design parameters that could be tuned in order for the Bayesian decision-theoretic MAMS trial to have the desired frequentist properties.

The procedure used for tuning the design parameters is described in Appendix 1. Parameter values selected for the Bayesian decision-theoretic MAMS design were $n_{1}=144$ and *C*/*Q *=* *0.0015. Under the null scenario, neither experimental arm was declared superior to the control arm in 95.1% of trials. Average and median trial size under the null scenario were 206 and 180 and 90th and 95th percentiles were 324 and 360. Under the alternative scenario, arm 3 was declared superior to the control arm in 79.8% of the trials. Average and median trial size under the alternative scenario were 259 and 252 and 90th and 95th percentiles of trial sizes were both 396. We also evaluated the proportion of correct decisions for the selected Bayesian decision-theoretic MAMS design under a second alternative scenario θ=(0.20,0.35,0.35) where both experimental arms were superior to control. Under this second alternative scenario, both experimental arms were declared superior to control in 69.9% of the trials and at least one experimental arm was declared superior in 90.5% of the trials. Average and median trial size under this second alternative scenario were 263 and 252 and 90th and 95th percentiles were both 396.

We compared the proportion of correct decisions for the selected Bayesian decision-theoretic MAMS design to frequentist single-stage trials using Dunnett’s procedure and the adaptive two-stage procedure of Urach and Posch. The average trial size was equalized across designs using the same procedure as in simulation II. After equalizing the average trial size, the two-stage procedure of Urach and Posch declared arm 3 superior to the control arm under θ=(0.20,0.20,0.35) in 65.1% and 65.0% of the trials using O’Brien Fleming and Pocock-type boundaries, respectively. The single-stage design using Dunnett’s procedure declared arm 3 superior to the control arm in 60.3% of the trials. Under the second alternative scenario θ=(0.20,0.35,0.35), the two-stage procedure of Urach and Posch declared both experimental arms superior to the control arm in 58.5% and 60.5% of the trials using O’Brien Fleming and Pocock-type boundaries. At least one experimental arm was declared superior in 80.6% and 81.5% of the trials, respectively. Using a single-stage design with Dunnett’s procedure, 45.1% of the trials declared both experimental arms superior and 75.5% declared at least one arm superior. The lower proportions of correct decisions under the frequentist procedures observed under both alternative scenarios are in accordance with the results of simulation II and underline the power of the adaptive stopping procedure and decision-theoretic approach.

## 6. Discussion

We generalized the Bayesian adaptive decision-theoretic design for two-arm clinical trials proposed by Cheng and Shen^20^ to the setting of MAMS trials with *K* (K≥2) arms. We evaluated the frequentist operating characteristics of the method for trials with up to five arms and a binary outcome variable and made a comparison with nonadaptive single-stage trials and frequentist adaptive two-stage trials.

We found that our Bayesian adaptive designs correctly identified the best arm more often than single-stage clinical trials with same total sample size, both in the setting with and without a control arm. In the setting with a control arm, we found our Bayesian adaptive designs to outperform frequentist single- and two-stage procedures, with largest differences in the proportion of correct decisions occurring when both experimental arms were superior to the control arm. In the setting with only experimental arms, we found that adaptive dropping of arms further increased the proportion of correct decisions only when at least one of the arms was clearly inferior. In those settings it is beneficial to drop the inferior arms early such that more data can be collected in the other arms. However, when arms were similarly effective, the proportion of correction decisions sometimes decreased when allowing for early dropping. This is related to the unfortunate decision early in the trial to drop the best arm and can be prevented by accruing a larger number of patients in the first stage.

An attractive feature of our decision-theoretic framework is that it uses the expected reduction in loss as the single quantity to inform stopping of the trial. This is in contrast to standard frequentist and Bayesian MAMS designs that generally require monitoring of two separate quantities. In those designs early stopping for efficacy is guided by a frequentist test statistic or a quantile of the posterior treatment efficacy, whereas early stopping for futility is based on conditional power or posterior predictive probability of success at the end of the trial. In our simulations, we observed that adaptive stopping in our designs occurred both for reasons of efficacy and futility. In our simulation studies, we considered symmetric loss functions where all incorrect decisions result in the same loss. Our framework, however, also permits incorporation of more elaborate loss functions where the losses are different for false-positive and false-negative findings and vary across the experimental arms.

In the simulation study, we found that even for trials with five arms an acceptable proportion of correct decisions could be achieved while average trial sizes remained below 200 patients. Although we did not put a cap on the sample size in our simulations, all simulated trials ended after a finite number of stages. This is in accordance with a theoretical result derived by Cheng and Shen^20^ that states that for two-arm trials termination is achieved at finite study size with a probability of one. This means that even when the treatment efficacies are equal in the different arms, the study terminates at finite study size. In that situation, the proportion of correct decisions over replicated trials will be one over the number of arms when the different arms have equal priors. An additional simulation (not presented) showed that even when differences in efficacies tended to zero, expected total trial sizes for our designs remained quite stable. Nevertheless, one may consider putting a cap on the maximum number of stages in order to rule out very large studies and restrict total costs and duration of the trial. We illustrated the use of the cap in a practical example. Note also that both with and without specification of a cap, our procedures did not become computationally challenging, even not for settings with five arms. The computation time of our methods is determined by the number of times that an expected change in loss needs to be evaluated and therefore increases linearly with the number of stages, whereas the computation time of a backward induction procedure increases exponentially with the number of stages.^19,24^

Clinical trials involving human subjects are usually classified as phase I to IV trials. The four phases correspond to safety assessment, identification of effective drugs, confirmation of a drug’s efficacy and post-approval research. Our methods can improve efficiency of phase II trials by facilitating screening of multiple experimental drugs in a single trial. Another application is in phase IV trials in which multiple, approved drugs are compared and interest is in selecting a single drug that minimizes the expected loss in future patients. Bayesian approaches are not widely used in the setting of phase III trials because current guidelines for such trials require stringent control of the type I error. However, it has recently been recommended to shift the focus in phase III trials towards error rates that are more insightful than the familywise error rate, for instance, through use of decision-theoretic approaches that incorporate losses for incorrect decisions where the incurred losses depend on the seriousness of the incorrect decision.^32^ Based on this, we think that our approach for comparing two experimental arms to a control arm, where parameters are tuned such that the type I error is controlled, is a sensible and feasible alternative to larger nonadaptive phase III trials.

Although the methods presented are very general, we made some specific decisions regarding the settings of our simulation studies. Firstly, we considered the primary outcome to be dichotomous. The framework can however also be applied to continuous outcomes. Secondly, we assumed that the outcomes of all included patients are available when the decision to continue or stop the trial is made. This is a common assumption in adaptive trials literature with early outcomes like disease progression or recurrent disease. It must be noted that our approach can still be applied when there is a delay between the time of inclusion of the patient and the time of outcome acquisition, but the benefit of using an adaptive design instead of a nonadaptive design becomes smaller when the delay increases. In such settings, the posterior predictive distributions are based on the data observed up to time of the interim analysis. Finally, we used uninformative, uniform priors for the success probabilities in all simulations. The Bayesian approach facilitates the incorporation of historical data by means of an informative prior distribution, such as for instance a power prior.^33,34^ Especially in settings where an already widely studied standard treatment serves as a control, efficiency may be increased by incorporation of information obtained in earlier studies.

## Appendix 1

### A.1. Design of Bayesian decision-theoretic MAMS trials in R

R functions for evaluating the frequentist operating characteristics of Bayesian decision-theoretic MAMS trials can be found on https://github.com/PeterMVanDeVen/Decision-Theoretic-MAMS-trials. The R functions can be used in the design phase to compare the proportion of correct decisions for different values of *θ* and different choices for the design parameters. Here we describe the procedure used to tune the size of the initial stage *n*_1_ and the relative costs *C*/*Q* of the practical example. The procedure consisted of the following steps:

- We restricted our search to values for *n*_1_ that were multiples of the batch size n = 36 used in subsequent stages, implying a maximum number of 396 patients to be included.
- We first checked whether both the type I and type II error rate for a single-stage design with maximum trial size were below the target levels of 5% and 20%. If the type I error rate is above the target level, then the maximum trial size or the margin *δ* should be increased. If type II error rate is above the target level, then the maximum trial size should be increased, difference between response rates under the alternative scenario should be increased or the margin *δ* should be decreased. At the maximum trial size of 396, we observed type I and type II error rates of 3.3% and 18.9%, respectively.
- We simulated trials with early dropping but with a fixed total trial size of 396 under the null scenario θ=(0.20,0.20,0.20). We subsequently set *n*_1_ at the batch size 36 and its multiples 72, 108,… until the type I error rate was below the target level of 5%. At $n_{1}=144$, the type I error rate was 4.7%.
- We simulated trials with early dropping but with a fixed total trial size of 396 and an initial stage of size $n_{1}=144$ under the alternative scenario θ=(0.20,0.20,0.35) and checked whether the type II error rate was below the target level of 20%. At $n_{1}=144$, the type II error rate was 17.0%.
- To incorporate adaptive stopping, we reevaluated the simulated trials with $n_{1}=144$ using a fine grid of values for *C*/*Q*. This did not require additional simulations as the expected increases in the posterior probability of a correct decision for all subsequent stages until the maximum sample size had already been stored. We selected the maximum value of *C*/*Q* for which both the type I and type II error rate were below the target levels, which was C/Q=0.0015.
- Frequentist operating characteristics of the design with $n_{1}=144$ and C/Q=0.0015 were validated in an independent simulation of 5,000 trials. Type I and type II error rate were 4.9% and 20.1%, respectively.
